# High-Performance Siamese Network for Real-Time Tracking

**DOI:** 10.3390/s22228953

**Published:** 2022-11-18

**Authors:** Guocai Du, Peiyong Zhou, Ruxianguli Abudurexiti, Mahpirat, Alimjan Aysa, Kurban Ubul

**Affiliations:** 1 School of Information Science and Engineering, Xinjiang University, Urumqi 830000, China; 2 Department of Information Security Engineering, Xinjiang Police College, Urumqi 830000, China; 3 Office of Educational Administration, Xinjiang University, Urumqi 830000, China; 4 Key Laboratory of Xinjiang Multilingual Information Technology, Xinjiang University, Urumqi 830000, China

**Keywords:** receptive field, channel pruning, real time, tracking

## Abstract

Target tracking algorithms based on deep learning have achieved good results in public datasets. Among them, the network tracking algorithm based on Siamese tracking has a high accuracy and fast speed, which has attracted significant attention. However, the Siamese tracker uses the AlexNet network as its backbone and the network layers are relatively shallow, so it does not make full use of the ability of the deep neural network. If only the backbones of target tracking are replaced, there will be no obvious improvement, such as in the cases of ResNet and Inception. Therefore, this paper designs a wider and deeper network structure. At a wider level, a mechanism that can adaptively adjust the receptive field (RF) size is designed. Firstly, multiple branches are divided by the split operator, and each branch has a different size of kernel corresponding to a different size of RF; then, the fuse operator is used to fuse the information of each branch to obtain the selection weights; and finally, according to the selection, the aggregation feature map is weighted. At a deeper level, a new kind of residual models is designed. The channel is simplified by pruning in order to improve the tracking speed. According to the above, a wider and deeper Siamese network was proposed in this paper. The experimental results show that the structure proposed in this paper achieves a good tracking effect and real-time performance on six kinds of datasets. The proposed tracker achieves an SUC and Prec of LaSOT of 0.569 and 0.571, respectively.

## 1. Introduction

Target tracking is an important branch in the field of computer vision which plays an crucial role in many applications such as video surveillance, human-computer interaction, and unmanned driving [1]. Target tracking aims to track a target almost without any a priori knowledge, i.e., by manually or automatically selecting the target of interest in the initial frame of the video sequence, and then predicting the target size and motion trajectory in the subsequent frame through the size and position of the target. Due to the influence of light intensity change, rapid movement, motion blur, and scale change [2], target tracking is still a great challenge.

**Figure 1:**
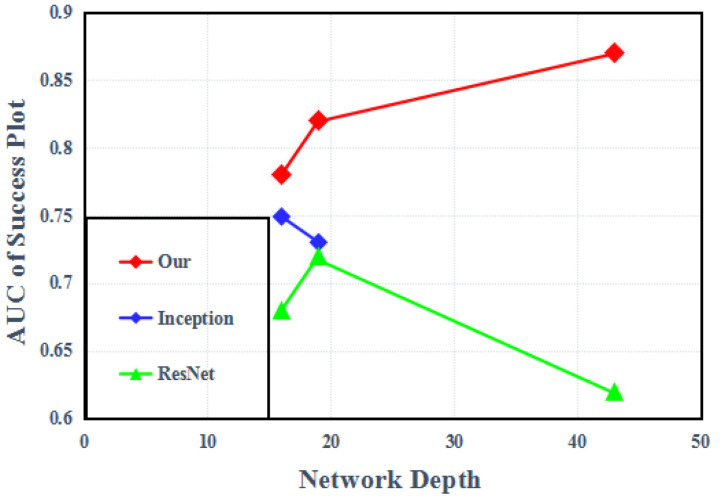
Impact of different network depths on tracking performance.

In recent years, the target tracking algorithm based on the Siamese network has shown its advantages in terms of high precision and fast speed, which have gradually attracted the attention of scholars. The representative Siamese algorithms are SiamFC [3], SiamMASK [4], and SiamRPN [5], etc. These algorithms learn the similarity metric between the template and candidate image patches through Siamese networks, and establish the target model as the search problem of the target in the whole image. These trackers have been using shallow neural networks rather than more complex networks. We find that if we replace the backbone with a broader and deeper network, such as VGG [6], Inception [7], and ResNet [8], then the experimental results are poor. Only replacing the network does not improve the tracking performance. On the contrary, when the width is very wide and the depth is very deep, the tracking performance decreases, as shown in (see Figure 1). The reason for this is that these wider and deeper network structures are more suitable for image classification, and target tracking involves a series of problems of accurate positioning which cannot be solved. By analyzing the structure of the Siamese network, it is found that the most important factors affecting the trackers’ tracking performance are the number of neurons, the number of channels, and the network stride. In particular, the receptive field (RF) determines which image region is used in computing a feature. A larger receptive field can provide a larger image context, but it is difficult for a smaller receptive field to capture the structure of the tracking target. The network stride will affect the output characteristics between the two layers, resulting in low-tracking accuracy. In addition, with the deepening of the network, the channel redundancy in the feature graph will lead to the consumption of a large number of storage and computing resources. These reasons make it difficult for Siamese trackers to develop into a wider and deeper network.

In this paper, a new residual structure was designed to solve the above problems. These modules can be developed into a wider and deeper network while the accuracy and speed are improved. Firstly, regarding the size of the receptive field, a wider network is designed to adaptively select the receptive field. Then, by pruning the channels, this kind of residual structure is stacked up to solve the problem that deeper Siamese trackers will encounter. At the same time, network stride is also an important factor that affects receptive fields. An excessively small network stride will affect the real-time tracking, while an excessively large network stride will also affect the accuracy of tracking. Through the comparison of the tracking experiments, it was found that the network stride was equal to 8, and the accuracy was higher. Finally, the experimental results show that the proposed method has a better performance than other state-of-the-art algorithms. More importantly, the new network architecture designed in this paper is of a lightweight level, which can achieve real-time performance in terms of speed.

There are three aspects in this paper:

Firstly, the inappropriate receptive fields will affect the ability to identify and locate, so the problem is solved to some extent by widening the network.

Secondly, since there is obvious redundancy between the different filters and characteristic channels, channel pruning is thus used to reduce the amount of computation. It is very effective in improving the tracking speed.

Thirdly, in order to improve the tracking accuracy, different network strides are set. When the network step size is 8, the tracking accuracy is higher. The experimental results show that the tracking accuracy and speed of the proposed network structure are improved.

The structure of this paper is mainly composed of the following. In the first section, the basic theory of target tracking is introduced, and then some problems as well as the solutions proposed in this paper are proposed and briefly analyzed. In the second section, Siamese tracking and attention mechanisms are reviewed, which provide the basic theory for the method proposed in this paper. In the third section, a new wide and deep Siamese network is proposed, which is implemented by adaptive receptive field and channel pruning, respectively. In the fourth section, we conduct a comparative experimental analysis of six different types of datasets as well as perform ablation experiments to prove the effectiveness of the method proposed in this paper. In the fifth and sixth sections, the work in this paper and related works are discussed. The last section is the conclusion.

## 2. Related Works

The improvement of this paper is mainly for the Siamese network, so this paper first briefly reviews two aspects related to the work of this paper: trackers based on a Siamese network structure and attention mechanisms.

### 2.1. Siamese Tracking

A Siamese network [9] is mainly composed of two branches. A pair of images is used as the input, which comprises exemplar image z and candidate search image x. Then, compare image z to image x through a function. If the two images depict the same, a high score will be returned; otherwise, a low score will be returned. Image z represents the target of interest. For example, this may be the image patch on the tracking target in the first frame of the video sequence. Image x is generally larger and represents the search area in the subsequent frame sequence. Both inputs need to be processed by a ConvNet φ with parameters θ. Then, two feature maps are generated and cross-correlated as

(1) $$f_{\theta }\left(z,x\right)=\varphi_{\theta }\left(z\right)\times \varphi_{\theta }\left(x\right)+b\cdot 1$$

where b·1 means the bias term and the value at each location b∈ℝ. Formula (1) is equivalent to image x performing an exhaustive search on image z. Finally, it aims to match the maximum value in response map f to the tracking target location.

In order to achieve this goal, the network is trained offline by random image pairs (z,x) and the corresponding ground truth label y in the training video. Parameter θ of ConvNet is obtained by minimizing the logistic loss l on the training set

(2) $$\arg\underset{\theta }{\min}\underset{\left(z,x,y\right)}{E}l(y,f_{\theta }(z,x))$$

### 2.2. Attention Mechanisms

In recent years, attention mechanisms [10] have been used in many fields, such as natural language processing, image segmentation, and image classification, and their advantages were gradually reflected in various fields. The attention mechanism can highlight the most abundant feature expression and inhibit the feature expression with weak information. In the direction of image classification, Wang [11] proposed to use trunk and mask attention in the middle stage of CNN, and introduced the hourglass unit to complete the global emphasis of the spatial and channel dimension.

Inspired by the anchor’s local receptive fields [12,13], we built the past continuous neural networks (CNNs), and then continued to build the modern CNN structure. This can help neurons collect multi-scale spatial information in the same processing stage. However, some of the RF characteristics of cortical neurons have not been paid attention to in CNN. For example, these include the adaptive changing of the RF size. The experimental results show that the visual cortex can adaptively adjust the RF size according to the size of the stimulus. However, this feature is not used to build many networks.

## 3. Wider and Deeper Siamese Network

### 3.1. Adaptive Receptive Field

In order to solve the RF size problem, this paper proposes a nonlinear method which can adaptively adjust the RF size for neurons. The split operator generates different paths according to different kernel sizes, which correspond to the different RF sizes of neurons. The fuse operator fuses different path information to obtain a global and comprehensive representation for selection weights. The select operator fuses the feature maps of kernels with different sizes through selection weights. In this paper, only two convolution kernels with different sizes are used, and more branches can be expanded.

**Split.** Firstly, for any feature map $X\in {\mathbb{R}}^{H^{\prime }\times W^{\prime }\times C^{\prime }}$, this will performed in two operations that transform $\tilde{F}:X\to \tilde{U}\in {\mathbb{R}}^{H\times W\times C}$ and $\hat{F}:X\to \hat{U}\in {\mathbb{R}}^{H\times W\times C}$, and the core sizes are 3×3 and 5×5, respectively. Among them, $\tilde{F}$ and $\hat{F}$ are composed of efficiently grouped (depth wise conversions) BN and ReLU functions. In order to accelerate the efficiency, the traditional convolution with a 5×5 kernel is replaced by the differentiated convolution with a 3×3 kernel and division size 2.

**Fuse.** According to the second section, in order to adaptively adjust RF sizes according to the stimulus content, the following steps must be followed. Firstly, the information flows of multiple branches are controlled by gates, which contain the information of different characteristics and enter into the neurons of the next layer. Then, the gates integrate the information of the branches, that is, the information of multiple branches is fused by element wise summary.

(3) $$U=\tilde{U}+\hat{U}$$

Finally, global average pooling is embedded into global information to obtain channel-wise statistics as $s\in {\mathbb{R}}^{C}$. The specific operation is as follows: compress U by spatial dimensions H×W to obtain the c element of s.

(4) $$s_{c}=F_{gp}(U_{c})=\frac{1}{H\times W}\sum_{i=1}^{H}\sum_{j=1}^{W}U_{c}(i,j)$$

Through a fully connected layer, we can improve the efficiency and reduce the dimension, and thus obtain a compact feature $z\in {\mathbb{R}}^{d\times 1}$.

(5) $$z=F_{fc}(s)=\delta (B(Ws))$$

Among them, z can provide guidance for adaptive selections, δ is a ReLU function, B is BN, and $W\in {\mathbb{R}}^{d\times C}$.

**Select.** Under the action of the compact feature descriptor z, soft attention across channels is used to adaptively select different spatial scales of information.

(6) $$a=\frac{e^{A_{c}z}}{e^{A_{c}z}+e^{B_{c}z}},b=\frac{e^{B_{c}z}}{e^{A_{c}z}+e^{B_{c}z}}$$

Among them, $A,B\in {\mathbb{R}}^{C\times d}$, a and b are soft attention vectors for $\tilde{U}$ and $\hat{U}$. $A_{c}\in {\mathbb{R}}^{1;\times d}$ are the c line of A, $a_{c}$ is the c element of a, and $B_{c}$ and $b_{c}$ are the same. Since $a_{c}+b_{c}=1$, matrix B is redundant when there are two branches. Through the attention weights of different cores, the final feature map V is obtained.

(7) $$V_{c}=a_{c}\cdot {\tilde{U}}_{c}+b_{c}\cdot {\hat{U}}_{c},a_{c}+b_{c}=1$$

Among them, $V=[V_{1},V_{2},\ldots ,V_{C}],V_{c}\in {\mathbb{R}}^{H\times W}$. In this part, only two branches are given, and the formula can be extended to obtain a wider network.

### 3.2. Channel Pruning

Through the first section, we find that the deepening of the network is accompanied by the extraction of useless features. Therefore, this paper proposes a new module, prune units, to eliminate the redundancy between different filters and characteristic channels. In this paper, channel pruning is used to reduce the computing cost of deep network layers. In neural networks, compared with other weight reduction methods, channel pruning is a naturally structured method, which does not introduce sparsity. Finally, it can increase the depth of the network.

Channel pruning is divided into two steps: channel selection and feature map reconstruction. In the first step, representative channels are selected according to LASSO regression, and useless channels are pruned. The second step is to reconstruct the new feature map through the minimum mean square error.

(8) $$\begin{array}{l} \arg\min_{\beta ,W}\frac{1}{2N}{\left\|Y-\sum_{i=1}^{c}\beta_{i}X_{i}W_{i}^{T}\right\|}_{F}^{2}+\lambda {\left\|\beta \right\|}_{1}\\ subject\;to{\left\|\beta \right\|}_{0}\leq c^{\prime },\;\;\;\;\forall i{\left\|W_{i}\right\|}_{F}=1 \end{array}$$

where N is the batch size, Y is the output characteristic graph, matrix of size N×n, X is a matrix of $N\times k_{n}\times k_{w}$, W is a matrix of $n\times k_{n}\times k_{w}$, Y is a matrix of N×n, β is a one-dimensional vector, and λ is a penalty efficient. With the increase in λ, the 0 item in β gradually increases, and the acceleration ratio is improved. The $\forall_{i}{\left\|W_{i}\right\|}_{F}$ of avoids a trivial solution. ${\left\|\right\|}_{F}$ is the Frobenius norm. $c^{\prime }$ is the number of channels after pruning. The solution of β,W corresponds to the channel selection and reappearance feature map, respectively.

**Figure 2:**
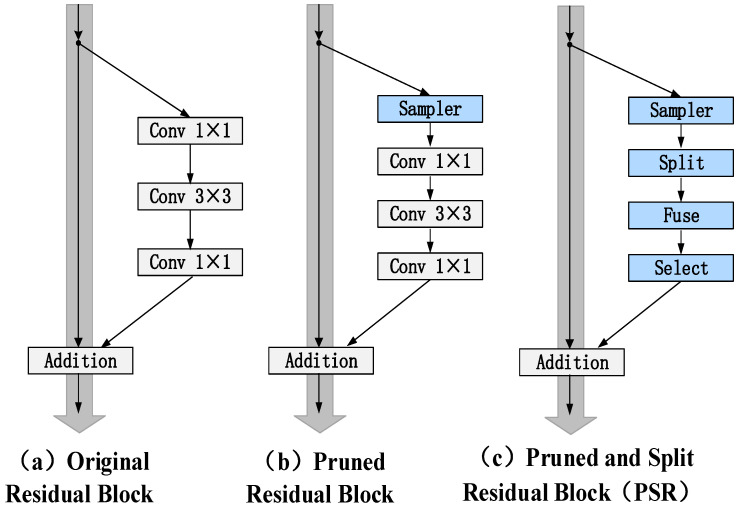
The proposed residual units. (**a**) represents the original residual block. (**b**) represents the pruning of the residual block. The input channel of the first convolution layer is sampled, and the rest is operated according to Section 3.2. (**c**) means to extend the width of the residual block. The specific operation is also in accordance with Section 3.2. The blue square indicates the difference between the proposed method and the original residual block.

For a single network (such as AlexNet [14], LeNet [15], and VGG), any convolution layer can be pruned by the above formula. However, it is not suitable for multi-branch ResNet. Thus, some changes were made to ResNet. The network is composed of a residual branch and shortcut branch. The residual branch consists of three convolutions. Firstly, for the first convolution layer, if it is pruned directly, the input feature map will be affected. At the same time, the input feature graph is shared with a shortcut, so it cannot be pruned directly. Therefore, the solution of this paper was to add a sampling layer, so that the original input feature graph can be retained without affecting the original input feature graph as the input of the shortcut, as shown in (see Figure 2b).

### 3.3. Build a Wider and Deeper Siamese Network

In this paper, a wider and deeper network can be built by adaptive RF and channel pruning. The residual block has an expression function that is easy to optimize and enhance, which is used in many network architectures (Table 1). It is mainly composed of two parts, namely three-stacked convolution layers and a shortcut connection that bypasses the three layers. The three layers are, respectively, 1×1, 3×3, and 1×1 convolutions, as shown in (see Figure 2a). Among them, 1×1 layers represent the reduced dimensions and restoring dimensions, and 3×3 layers represent the smaller bottleneck of the input and output dimensions. The overall improvement method is shown in Figure 2. This paper presents four deep prune and split residual networks, i.e., PSResNet-16, 19, 22, and 43. 

## 4. Experiments

This section uses six datasets for tracking performance comparison, which are compared with several state-of-the-art algorithms. Through the analysis of tracking accuracy, robustness, and real-time performance, the experimental results show that the proposed network structure is reliable.

### 4.1. Datasets and Evaluation Metrics State-of-the-Art Comparison

Visual object tracking (VOT) competition is one of the most important competitions in the field of target tracking organized by the vision team of the University of Slovenia. Since VOT-2013, competitions have attracted the attention of more and more researchers. Every year, VOT competitions propose new target tracking datasets to evaluate the most advanced trackers. The VOT dataset also contains a variety of complex scenes, marking visual attributes such as occlusion, lighting changes, motion changes, and scale changes by frame. VOT-2018 [16] contains 60 video sequences. VOT-2019 [17] is generated by replacing 20% of the less challenging video sequences in VOT-2018. VOT-2020 has 60 high-quality video sequences, and the dataset also has real-time testing. This paper uses VOT-2018, VOT-2019, and VOT-2020 [18] datasets.

The object tracking benchmark (OTB) [19] dataset is the most widely used dataset in the field of target tracking in recent years, proposed by Wu Yi’s team at the University of California. This article uses the OTB100 dataset, which contains 100 videos that have all been tagged. It uses a rectangular bounding box to initially tag the target. In order to better analyze the advantages and disadvantages of the tracker, OTB classifies the attributes of videos in different scenes. A video can have multiple attributes at the same time, which mainly consist of 11 types.

LaSOT [20] is a long-term tracking dataset jointly proposed by Temple University, South China University of Technology, and Pengcheng Laboratory in 2019. It consists of 1400 sequences, comprising more than 3.5 million images. The shortest video has 1000 frames, and the longest one contains 11397 frames. The average length of each video is approximately 2500 frames. The goals in the video are divided into 70 categories. Each category has 20 video sequences. Each video contains different challenges.

GOT-10k [21] is a large target-tracking dataset based on WordNet. It contains more than 10000 videos and tracking objects in real environments, including more than 560 categories and 87 motion modes. Each video contains two labels, namely category and motion mode, and the total number of bounding boxes is more than 1.5 million. This makes up for the shortcomings of having too few OTB and VOT categories at an excessively small scale. At the same time, GOT-10k provides target visibility ratio labels. Thus, the tracker’s perception of target motion and occlusion is improved. GOT-10k introduced the one-shot protocol for tracker evaluation for the first time. The categories in its training dataset and test dataset are zero overlap, avoiding the bias of evaluation results to familiar categories, and promoting the generalized development of trackers. There are 420 video sequences in the test set, including 84 object categories and 31 motion categories, reflecting the objects in the real scene and making the experiment closer to the real evaluation.

In this paper, six datasets are used for tracking performance comparison, namely VOT-2018, VOT-2019, VOT-2020, OTB-100, LaSOT, and GOT-10K. The expected average overlap (EAO), accuracy (A), and robustness (R) are used to evaluate the tracking performance. The EAO is calculated by calculating the average value of the corresponding overlap rate of the tracker in a large number of sequences with different lengths. The robustness measures the number of times the tracker loses the target during tracking. The tracker runs 15 times on each video sequence to reduce the randomness of the results. The robustness is calculated by averaging the ratio of lost frames from different runs. The OTB-100 dataset contains 100 video sequences, and the tracking algorithm is compared and analyzed by two indicators, namely precision (Prec) and area under the curve (AUC). The LaSOT dataset has longer video sequences, with an average of 2500 frames per video sequence. In this dataset, success (SUC) and precision (Prec) are used to compare and analyze the algorithms. The GOT-10K is a large dataset containing 10,000 videos. The online server is used to compare 180 video test sets. The average overlap (AO) and success rate (SR) were used as performance indexes. AO is the average overlap between the tracking prediction box and the truth value box. SR refers to the percentage of the number of successful tracking frames out of the total number of video frames when the overlap rate exceeds a certain threshold.

### 4.2. State-of-the-Art Comparison

**Figure 3:**
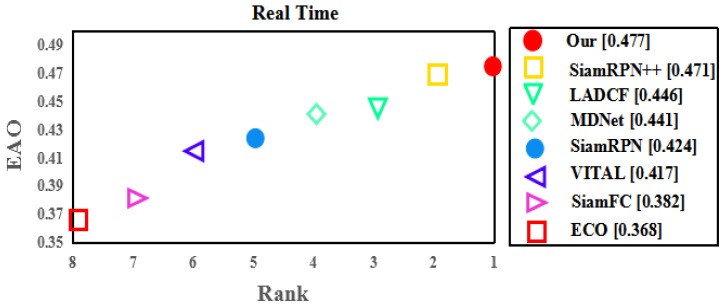
State-of-the-art evaluation on VOT-2020.

**Figure 4:**
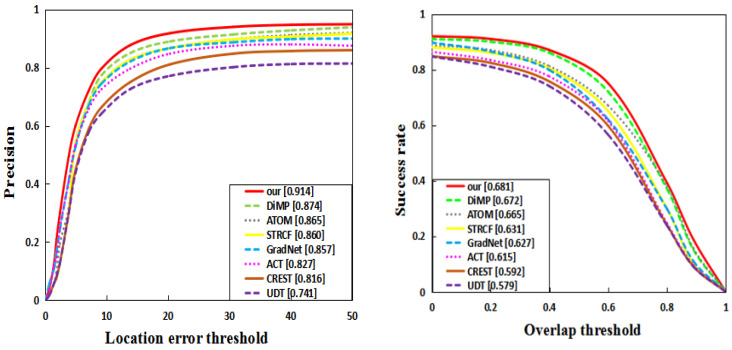

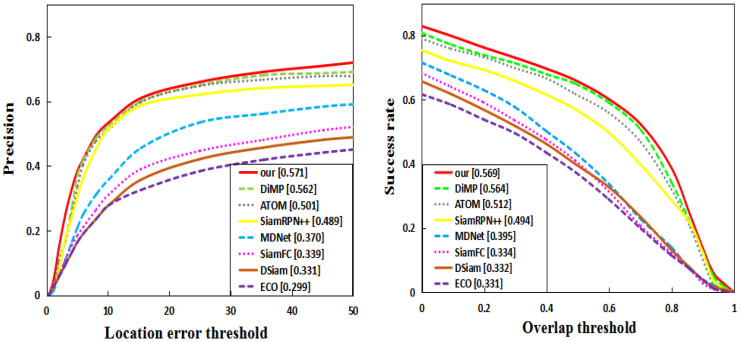
Success plot and precision plot of OTB-100 (**Top**) and LaSOT (**Bottom**).

**Figure 5:**
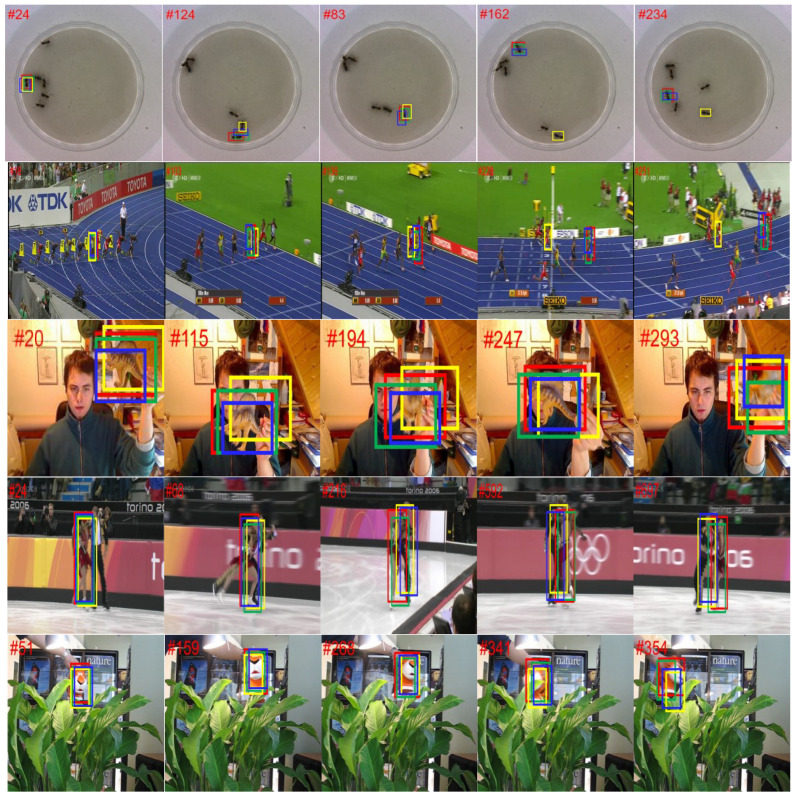

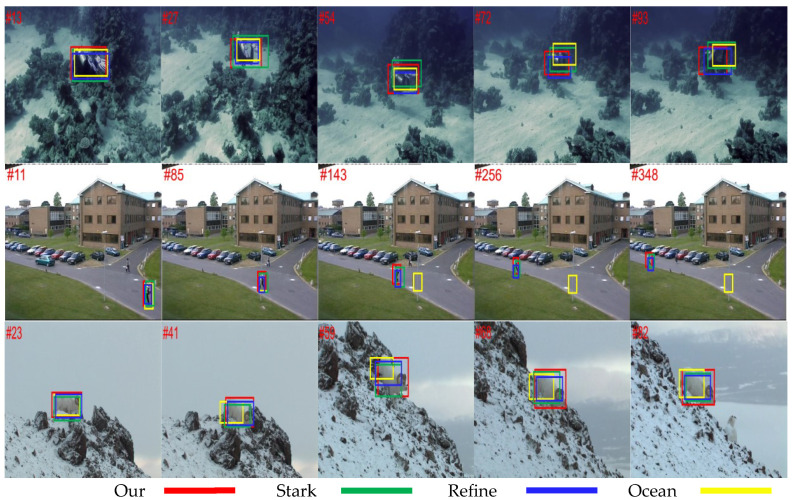
The qualitative results on five challenging sequences.

In order to evaluate the proposed method fairly, this paper compares the proposed method with 18 state-of-the-art algorithms, which include the representative algorithms in recent years. These are Refine [22], Stark [23], SiamFC [3], GradNet [24], C-RPN [25], TransT [4], SiamRPN++ [5], ATOM [26], ECO [27], UDT [28], CFNet [29], SiamRPN [5], LADCF [30], SiamRCNN [31], CREST [32], STRCF [33], ACT [34], MDNet [35], VITAL [36], and DaSiam [37]. The experimental results are shown in see Table 2, Table 3 and Table 4 (and see Figure 3, Figure 4 and Figure 5), respectively.

**VOT-2018 results.** This method was tested on the VOT-2018 dataset and compared with eight state-of-the-art methods. The dataset is one of the online model free single target trackers which contains 60 video sequences with different challenge factors. By using EAO, A, R, and AO (no-reset-based average overlap), the different algorithms are compared and analyzed. The experimental results are shown in Table 2.

**VOT-2019 results.** As can be seen in Table 3, the experimental results show that the proposed method achieves the best results. This experiment is a comparative analysis between VOT-2019 and recent popular algorithms. Compared with ATOM and EAO, this represents an increase by 7.8 and 0.6 points, respectively. In terms of robustness, this method is also optimal.

**VOT-2020 results.** As can be seen from Figure 3, the real-time performance of this method is better than those of other algorithms. It also has good tracking performance. Compared with SiamRPN, this method improves by 5.3 points and 3.6 points compared to MDNet, and the real-time performance is the best.

**OTB-100 results.** On the OTB-100 dataset, the algorithm is compared with other state-of-the-art trackers. Figure 4 shows that this method is superior to other methods. The experimental results show that the network architecture designed in this paper is effective. In addition, compared with the seven other algorithms, the proposed method has better precision and speed.

**GOT-10K results.** Table 4 shows the experimental results of this algorithm compared with other algorithms on the GOT-10K dataset. The AO score of the state-of-the-art trackers is 0.613, which is better than those of other methods. In the evaluation index of AO, the algorithm in this paper is 9.7 points higher than SiamMASK. The success rate is 0.8 points higher than SiamRCNN.

**LaSOT results.** In order to fully prove the effectiveness of this method, comparative analysis was carried out on LaSOT, and the experimental results are shown in Figure 4. A longer LaSOT video makes the dataset more challenging. The experimental results are shown in Figure 4. The SUC of this method is 0.569, while SiamFC is 0.334. Compared with ATOM, this paper improves by 5.7 points. These results show that the proposed method still has a good tracking ability for long video sequences.

### 4.3. Ablation Study

Ablation experiments were conducted on the dataset VOT-2020, and we evaluated the impact of different factors in the network on the target-tracking performance, as shown in Table 5 and Table 6.

**Impact of receptive field and stride.** Different networks are designed to evaluate the impacts of different factors on target tracking. The specific description is shown in Table 5. The different sizes of the stripe and receptive field have an impact on tracking performance. By constantly adjusting the size of these factors, we can specifically judge the impact on tracking performance. First, change the size of the convolution kernel in the residual network to control the receptive field. Taking Resnet-22 in Table 5 as an example, we can adjust the receptive field from +24 to −24, and obtain the best receptive field of +16. However, when the number of network layers changes, the best receptive field also changes. The network stripe can be changed by directly setting the parameters. It can be concluded from these that with the increase in RF, the performance will also be significantly reduced, and different RF sizes have their own network layers. For example, in Table 5, when the number of network layers is 16, the tracking performance is best when the RF is +8. When the number of network layers is 43, the RF is −16. Thus, an adaptive RF size adjustment is very important. The reason is that with the increase in RF, the area of the covered image is also increasing, which will lead to the extraction of useless features and the reduction in sensitivity to spatial positions. Then, an excessively large size of the network stripe will also affect the tracking performance. Setting it to 4 or 8 will be more stable and conform to the design size at the beginning of this article.

**With vs. with and with vs. without channel pruning.** Channel pruning is a very important part of our network structure. In order to reasonably evaluate its impact, we cut through a channel, i.e., Table 6 (setting 1) and a table without channel pruning, i.e., 6 (setting 2). Through table B, it is found that the performance has decreased to some extent within the acceptable range, however, the speed significantly improved. For example, the performance of channel pruning of Resnet-22 decreased from 0.284 to 0.282, a decrease of 0.2 points. In terms of speed, this increased from 69 (FPS) to 83 (FPS). This also clearly reflects the importance of channel clipping in improving the tracking speed. In order to more accurately analyze the effectiveness of our proposed method, we added a group of experiments (Setting 3). We compare the different settings in the network: (1) through the basic downsampling residual unit (Setting 3); and (2) our proposed channel pruning method (Setting 1). The experimental results show that our proposed channel pruning method is indeed effective. Simply using downsampling will not improve tracking. The reason is that it will remove the important features of the target, resulting in information loss.

## 5. Discussion

**Network Architectures.** The method proposed in this paper is mainly a module task designed based on the network architecture, which can be used not only for target tracking tasks but also for other tasks, such as target detection and multi-target tracking. The innovation of this paper is two-fold: depth and width. One is to make the network deeper, and the other is to make the network wider, whilst at the same time ensuring real-time tracking. In terms of network depth, ResNet’s design makes the network deeper through identity mapping, making ultra-deep networks possible. In terms of network width, GoogleNet’s design widens the width of the network through feature transformations in order to enhance the representation capacity of the target. The high-performance Siamese network proposed in this paper mainly solves two problems: one is the adaptive RF adjustment and the other is channel pruning—both of which affect tracking performance. Specifically, a mechanism that can adaptively adjust the RF size is designed from the network width. Firstly, multiple branches are divided by split operator, and each branch has a different size of kernel, corresponding to the different size of RF. Then, the fuse operator is used to fuse the information of each branch to obtain the selection weights. Finally, according to the selection weights aggregation feature map, design the channel pruning. The channel is simplified by pruning in order to improve the tracking speed. According to the above, a wider and deeper Siamese network was proposed in this paper. More importantly, there are few ways to design a network so that the tracking performance and time still have advantages when the number of layers and the width of the network are large.

**Siamese Trackers.** The Siamese trackers learn the similarity metric between the template and candidate image patches through Siamese networks. The initial work comprises SINT and SiamFC, both of which are based on the similarity matching strategy. Subsequently, a lot of work based on Siamese tracking was proposed. This is mainly divided into three parts. The first is the feature extraction method to accurately extract effective features for subsequent learning. The second part is to match the template features with candidate features. Generally, high-level semantic information and localization objects are used. The third part can enhance the feature of the target through online learning to prevent the loss of the target in the process of subsequent frames. In contrast to the above, our work analyzes the components of the network architecture, analyzes the importance of these components in tracking one by one, and then proposes an efficient network architecture. At the same time, the real-time performance of the tracking algorithm is guaranteed.

## 6. Conclusions

If the backbone is just replaced with other wider and deeper networks, the tracking effect will not be significantly improved. At the same time, with the increase in depth and width, the tracking performance will decrease significantly. Through the analysis, it was found that the RF and channel number are the main reasons for the degradation of the tracking performance. Therefore, this paper redesigns wider and deeper network architectures. This network is tested on six datasets, and the experimental results show that it can significantly improve the tracking performance.

## Figures and Tables

**Table 1:** Full architectures of the designed backbone networks for Siamese trackers. *G* denotes the grouped convolution. *M* denotes the number of paths.

Stage	PSResNet-16	PSResNet-19	PSResNet-22	PSResNet-43
Conv1	7 × 7, 64, stride 2			
**Conv2**	2 × 2, max pool, stride 2			$\left[\begin{array}{c} 1\times 1,\;64\\ PS\left[M=2,G=32,r=16\right]\\ 1\times 1,\;256 \end{array},64\right]\times 14$
	$\left[\begin{array}{c} 1\times 1,\;64\\ PS\left[M=2,G=32,r=16\right]\\ 1\times 1,\;256 \end{array},64\right]\times 1$	$\left[\begin{array}{c} 1\times 1,\;64\\ PS\left[M=2,G=32,r=16\right]\\ 1\times 1,\;256 \end{array},64\right]\times 2$	$\left[\begin{array}{c} 1\times 1,\;64\\ PS\left[M=2,G=32,r=16\right]\\ 1\times 1,\;256 \end{array},64\right]\times 3$	
**Conv3**	$\left[\begin{array}{c} 1\times 1,\;128\\ PS\left[M=2,G=32,r=16\right]\\ 1\times 1,\;512 \end{array},128\right]\times 4$	$\left[\begin{array}{c} 1\times 1,\;128\\ PS\left[M=2,G=32,r=16\right]\\ 1\times 1,\;512 \end{array},128\right]\times 4$	$\left[\begin{array}{c} 1\times 1,\;128\\ PS\left[M=2,G=32,r=16\right]\\ 1\times 1,\;512 \end{array},128\right]\times 4$	
	Cross-correlation Equation (1)			
**#Params**	1.201 *M*	1.247 *M*	1.316 *M*	1.143 *M*
**#FLOPs**	2.23 *G*	2.31 *G*	2.74 *G*	5.85 *G*

**Table 2:** Comparison with state-of-the-art trackers on VOT-2018 (**Red**, **Blue**, and **Green** represent the top three tracking effects. The bigger the EAO and A, the better the reverse R).

	ECO	DaSiamRPN	LADCF	Stark	ATOM	C-RPN	SiamFC	TransT	Our
EAO	0.274	0.421	0.367	**0.478**	0.394	0.269	0.276	**0.476**	**0.491**
A	0.481	0.591	0.509	**0.598**	0.587	0.551	0.485	**0.597**	**0.611**
R	0.292	0.169	0.264	**0.176**	0.213	0.310	0.287	**0.177**	**0.127**

**Table 3:** Comparison with state-of-the-art trackers on VOT-2019. (**Red**, **Blue**, and **Green** represent the top three tracking effects).

	GradNet	ECO	SiamMASK	SiamRPN++	ATOM	Refine	Stark	Our
EAO	0.271	0.272	0.278	0.291	0.299	**0.325**	**0.380**	**0.377**
A	0.492	0.479	0.586	0.578	0.588	**0.589**	**0.592**	**0.594**
R	0.495	0.492	0.459	0.448	0.407	**0.278**	**0.382**	**0.276**

**Table 4:** Comparison with state-of-the-art trackers on GOT-10k (**Red**, **Blue**, and **Green** represent the top three tracking effects and the larger the AO and SR, the better the tracking effect).

	UDT	SiamFC	C-RPN	ECO	SiamRPN	SiamMASK	Stark	SiamRCNN	Our
AO	0.352	0.364	0.376	0.389	0.487	0.516	**0.611**	**0.608**	**0.613**
SR	0.374	0.387	0.394	0.402	0.574	0.608	**0.717**	**0.712**	**0.720**

**Table 5:** Ablation over a different receptive field and stride.

Class	1	2	3	4	5	6	7	8
**Receptive field**	+24	+16	+8	−8	−16	−24	+16	+16
**Stride**	8	8	8	8	8	8	4	16
**ResNet-16**	0.23	0.24	0.27	0.25	0.24	0.21	0.18	0.17
**ResNet-19**	0.25	0.24	0.26	0.27	0.24	0.21	0.22	0.20
**ResNet-22**	0.28	0.29	0.27	0.25	0.21	0.20	0.28	0.26
**ResNet-43**	0.24	0.27	0.26	0.25	0.28	0.26	0.23	0.22
**PSResNet-22**	0.29	0.31	0.30	0.32	0.30	0.29	0.28	0.27

**Table 6:** Ablation over different channel pruning settings. The FPS represents speed.

	ResNet-16	ResNet-19	ResNet-22	ResNet-43	PSResNet-22
**Setting 1**	0.272 (89 FPS)	0.275 (87 FPS)	0.282 (83 FPS)	0.289 (78 FPS)	0.294 (88 FPS)
**Setting 2**	0.275 (85 FPS)	0.278 (78 FPS)	0.284 [69 FPS)	0.291 [61 FPS)	0.293 (76 FPS)
**Setting 3**	0.268 (90 FPS)	0.270 (88 FPS)	0.274 (81 FPS)	0.271 (77 FPS)	0.290 (85 FPS)

## Data Availability

The data used to support the findings of this study are available from the first author upon request.

## Abbreviations

Symbols and abbreviations	Full meaning
RF	Receptive field
SUC	Success
Prec	Precision
A	Accuracy
R	Robustness
AO	Average overlap
IoU	Interaction over union
FPS	Frame per second
SR	Success rate
EAO	Expected average overlap
f	Response feature
δ	ReLU function
W,Y	Matrix
β	Channel selection
W	Reappearance feature map
